# The impact of perceived life stress and online social support on university students’ mental health during the post-COVID era in Northwestern China: gender-specific analysis

**DOI:** 10.1186/s12889-024-17935-x

**Published:** 2024-02-14

**Authors:** Moye Xin, Chengxi Yang, Lijin Zhang, Chenzhuo Gao, Sasa Wang

**Affiliations:** 1 School of Psychology, Shaanxi Normal University, No. 199, South Chang’an Road, 710062 Xi’an, Shaanxi China; 2 Shaanxi Key Research Center for Children Mental and Behavioral Health, Shaanxi Normal University, Xi’an, China; 3 Shaanxi Key Laboratory of Behavior and Cognitive Neuroscience, Shaanxi Normal University, Xi’an, China; 4 College of Liberal Arts, Yulin University, No. 51, Chongwen Road, 719000 Yulin, China; 5 Department of sociology, School of philosophy, Shaanxi Normal University, No. 199, South Chang’an Road, 710062 Xi’an, China

**Keywords:** Mental health, Perceived life stress, Online social support, Post-COVID era, Gender differences

## Abstract

**Background:**

Before the pandemic, research had already established the potential impact of perceived life stress and social support on the mental health status of Chinese students. However, in the Post-COVID Era, the specific mechanisms linking these variables and the distinct role of online social support remain relatively unexplored.

**Methods:**

After the cessation of China’s dynamic zeroing policy, a total of 1180 university students from Northwestern China participated in this study by completing a demographic questionnaire, as well as self-report measures assessing mental health, perceived life stress, and online social support.

**Results:**

Approximately 25% of students exhibited psychological symptoms. When examining different categories of perceived life stress, males reported experiencing a significantly greater impact in terms of punishment and interpersonal relationships compared to females. Females experienced significantly higher levels of learning pressure compared to males. Specific types of perceived life stress were found to be significant predictors of students’ mental health status. Moreover, online social support was identified as a significant moderator in the relationship between all types of perceived life stress and mental health, irrespective of gender.

**Conclusion:**

Our study findings unveiled two significant aspects: Firstly, the impact of perceived life stress on the mental health of students was identified as a risk factor. Secondly, the role of online social support emerged as a protective factor, particularly in the post-pandemic context. Additionally, gender-specific patterns were observed in these relationships.

**Supplementary Information:**

The online version contains supplementary material available at 10.1186/s12889-024-17935-x.

## Introduction

The outbreak of the COVID-19 pandemic at the beginning of 2020 had severe impacts on society and the global economy in addition to disrupting people’s day-to-day life. Currently, global centres of epidemic prevention and control have entered a new stage of normalization in the “post-COVID era” [1]. During this post-COVID era, considered the time period following the most serious medical consequences of the pandemic, the psychosocial consequences of COVID-19 have begun to receive worldwide attention, especially individual perceived stress caused by the epidemic [2]. Perceived stress refers to the psychological, physiological, and behavioural stress that occurs in an individual’s living environment and has a significant predictive effect on the deterioration of mental health. Perceived life stress has been widely recognized as a significant risk factor for mental health problems, and numerous studies have highlighted the detrimental effects of high levels of stress on individuals’ psychological well-being. A study by Collins investigated the relationship between perceived life stress and symptoms of depression and anxiety in a large sample of adults. The results revealed a strong positive association between higher levels of perceived stress and increased symptoms of depression and anxiety [3]. These findings suggest that chronic exposure to stressors in daily life can significantly impact mental health outcomes. Another study focused on the effects of perceived life stress on the development of posttraumatic stress disorder (PTSD). The researchers found that individuals with higher levels of perceived stress were more vulnerable to experiencing PTSD symptoms after exposure to a traumatic event [4]. This highlights the importance of examining perceived stress as a potential risk factor for the development and exacerbation of mental health disorders. Furthermore, a longitudinal study by Babitha and colleagues explored the long-term consequences of perceived life stress on overall mental well-being during the COVID-19 pandemic lockdown period [5]. The findings indicated that sustained high levels of perceived stress over time were associated with an increased risk of developing chronic mental health conditions, such as major depressive disorder and generalized anxiety disorder. This study underscores the long-lasting impact of perceived stress on mental health outcomes. Overall, the reviewed research supports the notion that perceived life stress has a significant negative impact on individuals’ mental health. Previous studies highlight the association between higher levels of perceived stress and an increased risk of developing mental health disorders, worsening symptoms of existing conditions, and overall decreased psychological well-being. Acknowledging and addressing perceived life stress is crucial for promoting mental health and well-being.

University students with little social life experience, unstable psychological states, and high levels of emotional fluctuation reported lower psychological resilience during and after the pandemic, making them more prone to negative emotions (e.g., fear, anger, anxiety, restlessness) than other people [1]. Long-term psychological consequences (e.g., perceived life stress) can have an impact on individuals’ mental health [6]; however, there is insufficient research in this field in China. Some people adopt positive coping styles to address and relieve stress, which can promote mental health. Other people adopt negative coping styles, which may lead perceived stress to exceed a tolerable threshold, hinder mental health, and even lead to health risk behaviours [7]. There is agreement among the international academic community that online social support can alleviate the perceived life stress caused by the epidemic [8–10]; thus, its applicability among Chinese university students is worth discussing.

After the start of the new millennium, the popularity of internet-based social communication has increased, and has been used to exchange emotional and social support through online communication, which can create a sense of belonging [11]. The rapid development of technology has led to advances in communication that may affect how people perceive social support [12]. Online social support, which is the sense of identity and belonging that people gain when they feel understood and respected during emotional exchanges conducted through virtual platforms, also extends and expands traditional social support. Several studies have explored the impact of online social support on individuals’ sense of identity and belonging. For instance, Hua found that online communities provide a unique space for individuals to share personal experiences, seek advice, and receive empathy from others with similar interests or challenges [13]. The sense of identity and belonging cultivated through virtual platforms can enhance individuals’ emotional well-being and satisfaction with their social interactions. Online social support has also been shown to provide individuals with access to diverse sources of support. Researchers conducted a meta-analysis of studies on the effectiveness of online support groups and found that individuals who engage in online support networks reported increased social support regardless of their geographical location or demographic characteristics [14]. This finding suggests that virtual platforms have the potential to connect individuals with a wider range of support resources beyond their immediate physical environment. Furthermore, advances in technology have expanded the ways in which online social support can be offered. With the rise of social media platforms and mobile applications, individuals can now access support anytime and anywhere. This ubiquitous nature of online support allows for instant communication and the ability to reach out for help or provide support in real time [15]. Additionally, the integration of multimedia features such as video chats and virtual reality environments further enhances the richness and depth of online social support interactions [16]. Modern developments break the constraints of time and space and provide active and effective mental health education to students while ensuring safe social distancing. The unique characteristics of online support (e.g., nonphysical contact, privacy) can effectively buffer stressful negative psychological and stressful effects of the epidemic and can help students establish a healthy and positive attitude towards the post-COVID era.

However, some studies suggest that online social support may not always have a positive impact on mental health [17, 18]. The transient nature of online support may mean that the sense of respect and support provided through virtual communication does not effectively reduce pressure in the long run [19]. In fact, excessive reliance on online support groups could lead to internet addiction, where online social relationships become substitutes for in-person connections, especially when individuals feel unrecognized or unsatisfied in their daily lives [20]. Additionally, online social groups may expose individuals to antisocial personality-based groups that may promote health risk behaviours during online interactions [21]. Therefore, further investigations and additional studies are needed to determine whether online social support has a protective effect on the relationship between perceived life stress and mental health among university students.

As of June 2022, the number of netizens in Shaanxi Province reached 38.7 million, with 716,000 new netizens added throughout the year for a growth rate of 2.34%. In Shaanxi Province, the cumulative number of 5G base stations has exceeded 22,000, with more than 15.6 million households using 5G services. Moreover, the number of Internet users has exceeded 32 million, and the total length of optical cables in the province has surpassed 1.72 million kilometres. Urban communities and administrative villages have achieved dual coverage of fibre optic and 4G networks. The average download speed of fixed broadband networks is 51.76 Mbit/s, ranking second in the western region of China [22]. Among the professional structure of netizens, students in school account for the highest, number at 19.5%. The age distribution is mainly concentrated in the range of 19 to 24 years, accounting for 27.6% [23],. These findings indicate that young people, mainly university students, constitute the main group of internet users in the province. During the epidemic, the usage rate of online psychological counselling and support websites and apps also increased significantly (e.g., “one-on-one” online counselling for epidemic prevention and to address college students’ mental health was conducted by a university in Shaanxi). Furthermore, a study by Li revealed that university students who actively engaged in online social support groups experienced lower levels of emotional distress during the COVID-19 pandemic [24]. These groups provided a platform for students to share their concerns and receive empathetic responses from their peers, thereby reducing feelings of isolation. These social support strategies based on online platforms alleviated the perceived pressure and adaptive impact of the epidemic on the student population and reduced students’ risk of emotional and organizational dysfunction while providing communication and relieving the stress caused by the relatively closed environment of university students under lockdown [25].

Shaanxi Province is considered one of the major education provinces in China and has a large number of universities. During the epidemic, universities in Shaanxi Province faced challenges in coping with students’ mental health (e.g., the inability to manage students’ mental health face to face because of strict social distancing policies) [26]. It is therefore urgent to clarify the relationship between online social support and university students’ psychological status. This study attempts to answer the following research questions: do different types of perceived life stress have a definite impact on the mental health of university students in Shaanxi Province, China? Is there a gender difference in this effect? Does online social support have a direct or moderating impact on the mental health of university students facing perceived life stress in post-COVID era?

Meanwhile, previous studies have investigated gender differences in mental health, life stress, and online social support among university students. (1) Studies have explored gender differences in overall levels of perceived life stress among university students. Liu and Mustanski [27] reported that male students tend to experience greater levels of perceived life stress than female students do, supporting H1a. Similarly, Almeida and Piuvezam [28] observed greater stress levels among male students, indicating a consistent trend. (2) The impact of gender on mental health outcomes has also been investigated. Chinese researchers reported that male university students exhibited higher rates of mental health problems than their female counterparts [29], supporting H1b. These findings were further corroborated in a study by Li [30] that highlighted gender disparities in psychological distress during the COVID-19 pandemic. (3) Studies of gender differences in online social support among university students have presented varying results. Chyung [31] revealed that male students receive more online social support than female students do, supporting H1c. However, it is important to note that these findings contradict the prevailing notion that women typically receive higher levels of social support than men do [32]. (4) Specific types of perceived life stress can have a detrimental impact on mental health among university students, validating H2. Zhang highlighted the association between various stressors and worsened mental health outcomes, emphasizing the risks posed by high levels of stress [29]. (5) The link between online social support and mental health among university students has also been explored. Yong [33] identified a significant direct impact of online social support on mental health, lending support to H3. These findings highlight the potential of online platforms to provide valuable social support resources for students’ well-being. (6) Studies have investigated the moderating role of online social support in the relationship between perceived life stress and mental health among university students, supporting H4. Almeida and Hui [28, 34] found that online social support acts as a protective factor to buffer the negative impact of stress on mental health.

Existing literature substantiates the hypotheses delineated herein. Empirical research indicates gender disparities in perceived life stress, with males frequently reporting elevated stress levels. Moreover, males display greater vulnerability to mental health challenges. Notwithstanding the contention surrounding gender variations in online social support, evidence demonstrates their tangible effect on mental health and their potential moderating influence on the perceived life stress-mental health nexus. These insights highlight the significance of comprehending gender-specific patterns and the efficacy of online social support in fostering improved mental health among university students. Consequently, this study posits the following six hypotheses:

H1a: There are gender differences in overall statistics and specific types of perceived life stress, and male students may experience more perceived life stress than female students.

H1b: There are gender differences in mental health among university students, and male students may experience more mental health problems than female students.

H1c: There are gender differences in online social support among university students, and male students may receive more online social support than female students.

H2: Specific types of perceived life stress worsen mental health among university students and may represent a risk factor.

H3: Online social support has a significant direct impact on the mental health of university students.

H4: Online social support significantly moderates the relationship between perceived life stress and mental health among university students, indicating that it might be a protective factor.

**Fig. 1:**
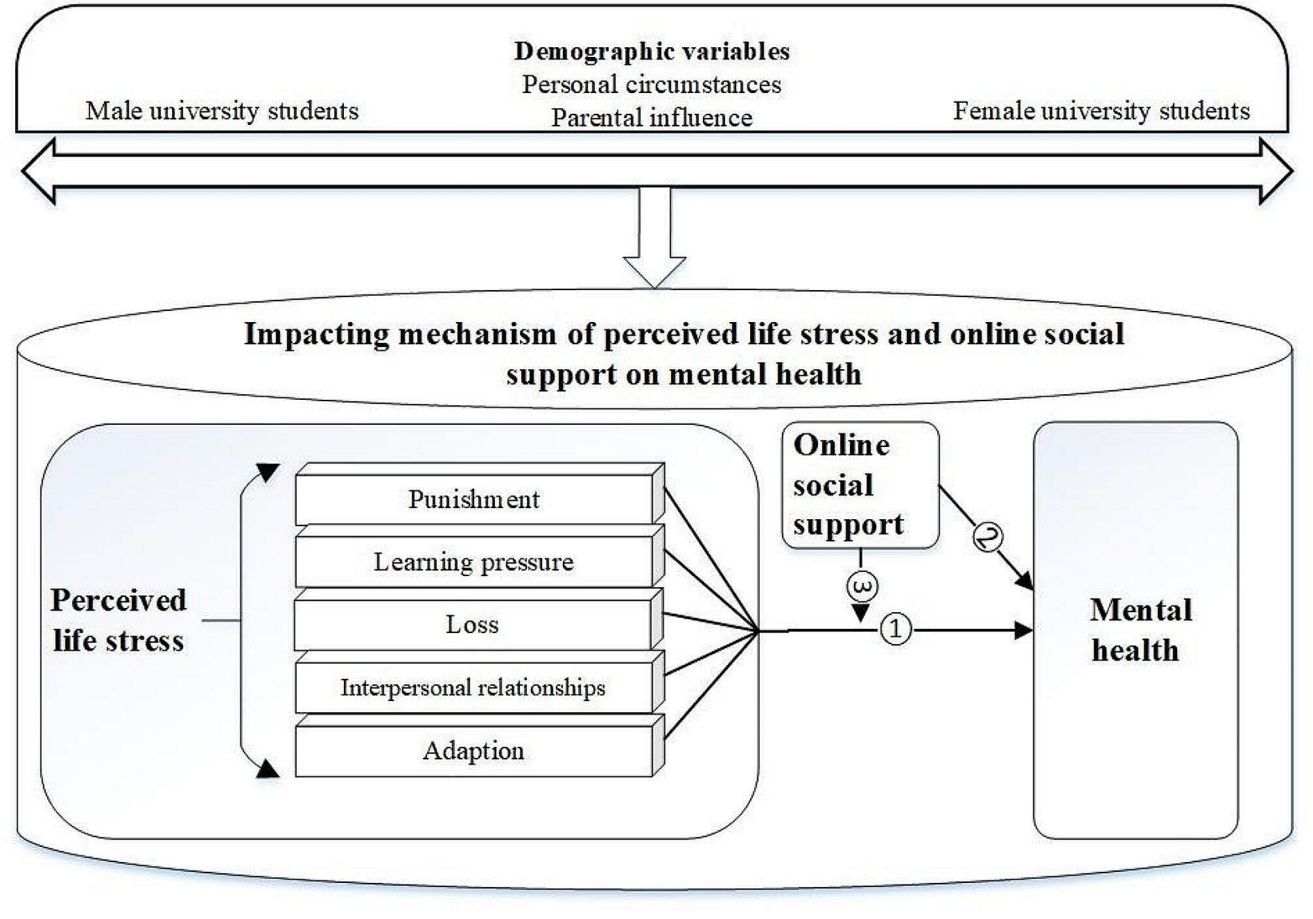
The direct and moderating effects of online social support on mental health (Path 2–3)The present study assessed four main research objectives corresponding to the research questions. Objective 1 was to assess potential gender differences in (**a**) mental health; (**b**) overall reports of perceived life stress and specific types of stress (i.e., punishment, learning pressure, loss, interpersonal relationships, adaptation); and (**d**) online social support among university students. Objective 2 was to determine whether specific types of perceived life stress impacted mental health among university students (Fig. 1, Path 1). Objective 3 was to determine whether online social support had direct effects on mental health among university students (Fig. 1, Path 2). Objective 4 was to determine whether online social support could significantly moderate the relationship between perceived life stress and mental health among university students (Fig. 1, Path 3) among university students under perceived life stress (Path 1)

## Materials and methods

### Data collection

The data for this study was obtained from the “Health Risk Behavior Survey of University Students” conducted in Xi’an, Yulin, and Ankang cities of Shaanxi Province in February and March 2023, following China’s cessation of the dynamic zeroing policy. The study recruited interested students from three universities in the region. With the cooperation of local school institutions and teachers, responsible teachers and student representatives were gathered. The investigators first explained the principles of privacy protection to the responsible teachers and obtained consent from the college students. They were also informed that they could withdraw from the survey at any time if they experienced any physical or psychological discomfort. Afterwards, questionnaires were distributed to the responsible teachers at each university, who then distributed them to the students in their respective departments. The respondents had the option to fill out the questionnaire on their own or anonymously with the assistance of the investigators, and the completed questionnaires were collected immediately. In order to incentivize the cooperation of the respondents and ensure the smooth and complete collection of data, each participant received a reward of 20–30 RMB from the investigators after completing the questionnaire. Additionally, with the voluntary agreement of the participants, long-term psychological counseling and support were provided individually and privately. To ensure anonymity, all questionnaires were anonymous, and the survey commenced only after receiving approval from all participants. Each participant completed a self-administered physical questionnaire. This study excluded certain categories of university students: (1) Individuals with limited understanding and judgment abilities: If students are unable to comprehend the purpose and content of the research due to age, cognitive abilities, or other factors, their suitability for participation may be questioned. (2) Conflicts of interest: Students who have conflicts of interest related to the research project may compromise the objectivity and impartiality of their participation. (3) Physical or mental health concerns: Students who may encounter physical or psychological discomfort during their involvement in the research should be safeguarded against any additional distress. Thus a total number of 1,400 questionnaires were distributed, and 1,180 valid questionnaires were collected, resulting in a participation rate of 84.3%. Among the participants, there were 655 male students, accounting for 55.5%, and 525 female students, accounting for 44.5%.

### Measures

#### Perceived life stress

We used the modified self-rating perceived life stress scale (MSPLSS) to assess perceived life stress [35]. This scale consists of 27 items, which are categorized into six types of perceived life stress: interpersonal relationships (e.g., being misunderstood or wronged; experiencing discrimination or cold treatment), learning pressure (e.g., failing an exam), punishment (e.g., being criticized or punished at school), loss (e.g., sudden death of a relative or friend; experiencing theft or losing items), and adaption (e.g., transferring or suspending activities; major changes in daily routines).

For the purpose of this study, we adapted the original scale to better suit the age of our participants and to more accurately measure the relative impacts of perceived life stress. We conducted a principal component analysis and used the maximum variance method to rotate the extracted factors. We obtained a total of five factors, meeting the numerical requirements of the fitting index test without altering the original six-factor concept of the scale. The 5-factor model demonstrated improved fitting index results, with correlation coefficients ranging from 0.694 to 0.788 (*p* < 0.01) and enhanced structural validity.

Compared to the original 6-factor model, the 5-factor model of the scale excluded the “other types” factor while assessing five types of perceived life stress among adolescents and young adults: punishment, learning pressure, loss, interpersonal relationships, and adaption. Respondents were asked to rate each item on a 5-point Likert scale, indicating the extent to which each event impacted their life, ranging from 1 (no impact) to 5 (extremely severe impact). The modified ASSLEC demonstrated high reliability with a Cronbach’s alpha coefficient of 0.87.

#### Mental health condition

Symptom checklist 90 (SCL-90), which was compiled by DeRogatis [36], with 90 items including 9 factors (i.e., somatization, obsessive-compulsive symptoms, interpersonal sensitivity, depression, anxiety, hostility, terror, paranoia, and psychoticism), was divided into 5 grades (1–5). Grade 1 is none, grade 2 is mild, grade 3 is moderate, grade 4 is relatively severe, and grade 5 is severe. If the total score reaches 160 or any factor score reaches 2, the positive items are detected. The SCL-90 demonstrated excellent reliability throughout the survey (α = 0.93).

#### Online social support

To measure the extent to which participants obtained emotional and practical support through the Internet, we utilized the 23-item online social support scale developed by Liang [37]. Participants were asked to rate the degree of online social support they received on a 5-point Likert scale, ranging from 1 (none) to 5 (always). Online social support were divided into several types and measured based on the nature of the support provided in the current study: (1) Emotional Support: This type of support includes expressions of empathy, understanding, and encouragement. It aims to provide comfort, validation, and a sense of belonging to individuals experiencing emotional distress. (2) Informational Support: Informational support involves sharing knowledge, advice, or resources to address specific concerns or problems. It can include providing guidance, suggestions, or practical information to help someone make informed decisions or solve a problem. (3) Tangible Support: Tangible support refers to offering material or practical assistance to meet someone’s needs. It could involve providing financial aid, access to resources, or physical help in carrying out tasks or responsibilities. (4) Esteem Support: Esteem support focuses on boosting an individual’s self-worth, confidence, and self-esteem. It includes compliments, positive feedback, and recognition for achievements or qualities. (5) Network Support: Network support emphasizes expanding social connections and facilitating social interactions. It involves introducing individuals to others, creating opportunities for networking, and fostering a sense of community. These types of support are not mutually exclusive, and individuals may seek or receive multiple forms of support simultaneously or at different times, depending on their needs and circumstances. Thus the scale includes items (e.g., “How often do you receive words of encouragement or empathy from others online?“, “Rate the availability and usefulness of information you receive from online sources when seeking solutions to your problems.“, “How often do others online provide positive feedback or compliments regarding your skills or accomplishments?“, “Rate the extent to which online platforms help facilitate meaningful social interactions and create a sense of belonging.“, and “Compared to offline communication, do you prefer spending more time engaging in online communication because it is more reliable and relaxing?” etc.) These questions were adopted to measure the different types of online social support and evaluate their presence and impact on individuals within digital communities. To assess the overall level of online social support received, a mean score was calculated, where higher scores indicate greater levels of support. The scale used in this study has shown satisfactory reliability, with a Cronbach’s alpha coefficient of 0.835.

### Data analysis strategy

A chi-square test was performed with SPSS 20 to examine gender differences in mental health among university students. Additionally, a series of one-way ANOVAs were conducted to explore potential sex differences in perceived life stress and online social support.

Stepwise linear regression analysis was used to assess the associations between different demographic variables (Step 1), perceived life stress (Step 2), and mental health conditions (Model 1–2) for both genders. Specifically, Model 1–2 (M = males; F = females) treated mental health as the dependent variable. Model 1 included demographic variables such as age, only child status, and parents’ marital status, while Model 2 incorporated perceived life stress as the main independent variable. For detailed information, please refer to Fig. 1; Table 1.

**Table 1:** Specific information of related models and steps (hypothesis 2)

Dependent Variables	Models	Independent Variables
Mental health condition	Model 1 M (Step 1)	Demographic variables
	Model 2 M (Step 2)	Demographic variables (e.g., age、only child or not、parents’ marital status)+ Perceived life stress (e.g., punishment)
	Model 1-2 F	Same procedure as above

Note: M stands for “males”, F for “females”

To investigate Hypotheses 3 and 4, based on the research framework established by Hypotheses 1 and 2, we employed linear regression analysis to examine the direct and moderating effects of online social support on mental health among university students while considering perceived life stress for both male and female participants. A total of 6 models were evaluated. Model 3 (M/F) incorporated online social support (OSS) as an independent variable to assess its direct impact on the relationship between perceived life stress (independent variable) and mental health conditions (dependent variable). Following Model 3, interaction terms for different types of perceived life stress (punishment, learning pressure, loss, interpersonal relationships, and adaptation) were successively introduced in Models 4–8 to explore the potential moderating effects of online social support on mental health [38].

## Results

Table 2 data shows that 213 male students’ total SCL-90 scores exceed 160, indicating that 17.8% of male students have certain psychological symptoms affected by the epidemic. 312 students with obsessive-compulsive symptoms scored more than 2 points, indicating that 26.0% of male students may have obvious obsessive-compulsive symptoms, while 23.8% of male students with interpersonal sensitivity may have problems due to the impact of the epidemic, 22.4% of male students may have obvious depression tendency, 25.1% of male students may have serious anxiety tendency, and 25% of male students may have obvious paranoid tendency; However, the relative detection rates of somatization, hostility, terror and psychosis were slightly lower; When it comes to females, the manifestations of their psychological symptoms are basically similar to those of males, and the chi square test did not show significant gender differences (x^2^ = 1.37, *P* = 3.67).

**Table 2:** SCL-90 Detection rate of psychological symptoms

University Students (*N* = 1200)	Male (*N* = 655)	Percentage (%)	Female (*N* = 525)	Percentage (%)
Somatization	60	5.0	53	4.4
Obsessive-compulsive symptoms	312	26.0	303	25.3
Interpersonal sensitivity	285	23.8	293	24.4
Depression	269	22.4	257	21.4
Anxiety	301	25.1	313	26.1
Hostility	107	9.0	88	7.3
Terror	93	7.8	96	8.0
Paranoid	299	25.0	304	25.3
Psychosis	63	5.3	58	4.8
Total score	213	17.8	208	17.3

Note: +*p* < 0.1, *p < 0.05, **p < 0.01, ***p < 0.001

Results from one-way ANOVAs revealed significant gender differences in overall reports of perceived life stress (F *=* 9.198, *p* < 0.001) and online social support (F *=* 12.638, *p* < 0.001), whereby males all reported higher levels of both comparing to females (see Table 3 for details).

Moreover, analyses of gender differences across 3 specific types (i.e., punishment, learning pressure & interpersonal relationship) among 5 total types of perceived life stress, revealed that males could experience a significantly higher degree of perceived life stress related to punishment (F *=* 6.803, *p* < 0.001), and interpersonal relationship (F *=* 9.924, *p* < 0.01). Yet females only scored significantly higher than that of males in one specific perceived life stress, which is learning pressure (F *=* 2.777, *p* < 0.05). No significant gender differences emerged in reports of perceived life stress related to the category of loss (F = 5.277, *p* = 1.603) and adaption (F = 1.135, *p* = 1.368). Refer to Table 3 for detailed information.

**Table 3:** Descriptive analysis

	Male(*N* = 655)			Female(*N* = 525)		
	Min/Max	Mean(SD)		Min/Max	Mean(SD)	
Perceived life stress (Overall)	1/5	1.79(0.65)		1/5	1.67(0.57)	
	F = 9.198***					
Punishments	1/5	1.60(0.71)		1/5	1.36(0.51)	
	F = 6.803***					
Learning pressure	1/5	2.04(0.77)		1/5	2.18(0.69)	
	F = 2.777+					
Loss	1/5	1.63(0.62)		1/5	1.58(0.54)	
	F = 5.277					
Interpersonal relationship	1/5	2.18(0.74)		1/5	2.08(0.65)	
	F = 9.924**					
Adaption	1/5	1.85(0.71)		1/5	1.82(0.59)	
	F = 1.135					
Online social support (Overall)	1/5		2.71(0.73)	1/5		2.31(0.65)
	F = 12.638***					

Note: +*p* < 0.1, *p < 0.05, **p < 0.01, ***p < 0.001

Table 4 reported the results from regressive analysis on perceived life stress associated with mental health condition among university students. For male students, the results of models 1 M and 2 M revealed that only one specific type of perceived life stress had significant positive impact on their mental health: punishment (β = 0.376, *p* < 0.001). This indicated that increased reports of perceived life stress related to punishment was significantly associated with increased mental health problems among males. Demographic variables did not change the impact of punishment on male students’ mental health significantly, but the impact of adaption on mental health slightly increased (β *=* 0.167, *p* < 0.1). Parents’ marital status (i.e., remarriage (β *=* 0.193, *p* < 0.1), divorce (β *=* 1.162, *p* < 0.05)) had relatively significant impacts on males’ mental health condition.

For female students, models 1 and 2 F revealed a totally different pattern. Specifically, learning pressure *(*β *=* 0.289, *p* < 0.01), interpersonal relationships (β *=* 0.311, *p* < 0.001), and adaption (β *=* 0.186, *p* < 0.1) all emerged as significant positive predictors of females’ mental health condition. This indicated that increased reports of perceived life stress related to learning pressure, interpersonal relationships, and adaption were significantly related to increased mental health problems among females. Demographic variables have increased the impact of learning pressure and adaption on female students’ mental health (β *=* 0.332, *p* < 0.001; β *=* 0.198, *p* < 0.05) while the impact of other perceived life stress on mental health did not change significantly. Only age, parents’ marital status (i.e., remarriage, divorce) had significant impacts on females’ mental health condition (β *=* -0.148, *p* < 0.01; β *=* 0.298, *p* < 0.05; β *=* 0.173, *p* < 0.01).

**Table 4:** Impacts of different types of perceived life stress on mental health

Dependent variable: Mental health condition	Males (*N* = 655)		Females(*N* = 525)	
**Control variables** **(Demographic)**	Model 1 M	Model 2 M	Model 1 F	Model 2 F
Age	0.043	0.051	-0.146**	-0.148**
Studying period: University(Middle-highschool)	0.051	0.059	0.009	0.013
Only child or not: no(yes)	0.018	0.019	-0.007	-0.011
Parents’ marital status: Remarriage(First marriage)	0.191+	0.193+	0.294*	0.298*
Divorced(First marriage)	1.158*	1.162*	0.163*	0.173**
Widowed(First marriage)	0.036	0.038	0.021	0.028
**Independent variable**: perceived life stress				
Punishments	0.376***	0.388***	0.236	0.239
Learning pressure	0.019	0.021	0.289**	0.332***
Loss	0.044	0.046	0.083	0.088
Interpersonal relationship	0.013	0.018	0.311***	0.351***
Adaption	0.149	0.167+	0.186+	0.198*
Adjusted R^2^	0.133	0.236	0.136	0.248
F	25.671***	46.494***	39.267***	48.675***

Note: +*p* < 0.1, *p < 0.05, **p < 0.01, ***p < 0.001

As presented in Table 5 (Model 3 M), results revealed that online social support did not have a significant direct effect on mental health among male university students (β = 0.181, *p* = 0.343). The accumulation of online social support received by male students was not directly related to their mental health. However, with the addition of interactive variables (Models 4 M − 8 M), it was found that online social support had a significant negative moderating effect on the relationship between 3 types of perceived life stress and mental health among male university students (loss, interpersonal relationships, adaption, β = -0.426, -0.434, -0.488, all *p*’s < 0.001). In other words, online social support has reduced the impact of those types of perceived life stress on male students’ mental health condition as a protective factor. As presented in Table 5 (Model 3 F), results revealed that online social support did not had a significant direct effect on mental health among female university students as well (β = 0.163, *p* = 0.346). With the addition of interaction terms (Models 4-8 F), it was found that online social support had a significant moderating effect on the relationship between all types of perceived life stress and mental health among female university students (β = -0.513、-0.510、-0.500、-0.525、-0.523, all *p*’s < 0.001). Online social support also reduced the impact brought by all types of perceived life stress on female students’ mental health as a protective factor, similar to the results of male students.

**Table 5:** The direct and moderating effects of online social support on mental health condition among male and female university students

Independent variables: Mental health condition	Males (*N* = 655)							Females(*N* = 525)					
	Model 3 M	Model 4 M		Model 5 M	Model 6 M	Model 7 M	Model 8 M	Model 3 F	Model 4 F	Model 5 F	Model 6 F	Model 7 F	Model 8 F
Control variables													
Age	0.051	0.059	0.053		0.056	0.055	0.062	-0.139**	-0.284***	-0.280***	-0.277***	-0.279***	-0.270***
Schooling stages: University(Middle school)	-0.003	-0.005	-0.004		-0.003	-0.001	-0.006	-0.013	-0.019	-0.011	-0.013	-0.011	-0.011
Single child(not single)	-0.019	-0.025	-0.024		-0.025	-0.025	-0.024	-0.011	0.030	0.031	0.039	0.039	0.032
Marital status of parents: Remarriage(first marriage)	0.186+	0.198+	0.198+		0.198+	0.197+	0.199+	0.241*	0.253*	0.253*	0.251*	0.249*	0.249*
Divorced(first marriage)	0.162**	0.183**	0.188**		0.185*	0.184*	0.181*	0.117**	0.120**	0.118**	0.117**	0.114**	0.119**
Widowed(first marriage)	0.038	0.040	0.049		0.040	0.040	0.049	0.028	0.029	0.020	0.021	0.029	0.020
**Perceived life stress**													
Punishment	0.376***	0.383***	0.388***		0.441***	0.411***	0.493***	0.239	0.248***	0.041	0.038	0.041	0.059
Learning pressure	0.019	0.019	0.213***		0.311	0.313	0.314	0.332***	0.361**	0.367***	0.365***	0.363***	0.367***
Loss	0.044	0.135	0.138		0.287***	0.102	0.104	0.088	0.093	0.091	0.113***	0.098	0.094
Interpersonal relationships	0.013	0.018	0.019		0.114	0.123***	0.012	0.351***	0.383***	0.386***	0.383***	0.384***	0.384***
adaption	0.149	0.181	0.187		0.188	0.188	0.293***	0.198*	0.066	0.053	0.053	0.047	0.333***
Online social support	0.181	-0.014	-0.015		-0014	-0.058	-0.017	0.163	0.170	0.137	0.161	0.193	0.164
**Moderation analysis**													
Punishment * Online social support		-0.415							-0.513***				
Learning pressure * Online social support			-0.421							-0.510***			
Loss * Online social support					-0.426***						-0.500***		
Interpersonal relationships * Online social support						-0.434***						-0.525***	
adaption * Online social support							-0.488***						-0.523***
Adjusted R^2^	0.142	0.154	0.157		0.158	0.161	0.162	0.148	0.152	0.154	0.158	0.161	0.162
F	13.116***	14.557***	14.412***		14.529***	14.126***	14.646***	20.136***	22.323***	22.096***	21.429***	23.156***	22.647***

## Discussion

### Descriptive analysis discussion

Hypothesis one (H1a) was supported: a significant gender difference was found in the overall status of perceived life stress among university students across genders, and male students experienced more perceived life stress than female students, as hypothesized. This finding is consistent with related studies that claim that male students are more likely to be exposed to perceived life stress than female students, which could endanger their mental health [39]. Hypothesis two (H1b) was also supported: no significant gender difference was found for mental health among university students. This may be explained by the long-term Zeroing COVID and home quarantine policies implemented during the pandemic in China, which had a significant psychological impact on Chinese students under strict lockdown and caused anxiety, unease, panic and fear, regardless of gender [40]. Furthermore, Hypothesis three (H1c) was supported: a significant gender difference was identified in overall reports of online social support among university students across genders, with male students receiving more online social support than female students. This finding is consistent with one study indicating that when students encounter physical and psychological problems, male students tend to seek help from the internet, while female students prefer to seek help from offline or “real-life” sources [41, 42].

### Regressive analysis discussion

Hypothesis four (H2) was supported. Our results revealed that perceived life stress may act as a risk factor for university students’ mental health regardless of gender. It is noteworthy that specific types of perceived life stress (i.e., punishment, adaptation) were significantly correlated with male students’ mental health, while other types of perceived life stress had no significant impact on their mental health. These findings could be interpreted in terms of gender stereotypes, which are commonplace in Chinese culture [43, 44]. These stereotypes suggest that men exhibit greater psychological endurance and aggressiveness than women, who are generally considered gentle and mild. These gender stereotypes or gender biases have led to biased perceptions. For example, male students in China are exposed to incidents of punishment more frequently than female students [45], placing them at risk of mental health difficulties. Among university students who experienced long-term separation from family members, male students were more likely than female students to report mental health difficulties, a finding that is partly explained by male students’ relatively higher levels of introversion and neuroticism [46]. Male students showed less progress than female students in terms of intellectual development and environmental adaptability over time and were more likely to report more negative self-evaluations. These psychological factors represent risk factors that may lead male students to engage in risky behaviours [47]. However, no gender difference was found in the mental health of university students who spent more time living with their families [48].

Perceived life stress, specifically with regard to interpersonal relationships, learning pressure, and adaptation, has significant impacts on the mental health of female students. This finding can potentially be attributed to the deeply rooted perception of gender stereotypes or gender bias in traditional East Asian families [43]. Although this type of prejudice or bias is gradually diminishing due to socioeconomic and educational advancements, its effects persist and influence individuals [49]. Moreover, emerging literature suggests that gendered socialization practices continue to reinforce the notion that women tend to prioritize relational interdependence, which may amplify the effects of interpersonal relationships on their well-being [50, 51]. Recent research by Zhang has found that the perceived quality of friendships is more closely tied to psychological distress in women than in men, possibly due to the internalization of societal expectations that emphasize the importance of nurturing roles for women [52]. Additionally, a study by Santos indicates that these gendered expectations can affect cognitive processing related to social stimuli, potentially making interpersonal dynamics more salient for females [53].To alleviate the disproportionate impact on women’s well-being, it is imperative to engage in active restructuring of social norms and educational practices to value and reinforce a more balanced view of gender roles, encouraging emotional autonomy and resilience across all genders. It is also important to encourage critical thinking and media literacy to challenge stereotypes perpetuated through various channels. By addressing these psychosocial dimensions, strategies can be developed to foster gender equity and enhance the mental health outcomes for female students. It is also crucial to implement appropriate countermeasures to prevent mental health issues, particularly among female students who have recently encountered or experienced perceived life stress. These measures are essential not only for addressing mental health concerns but also for facilitating the elimination of gender bias.

In particular, students who face negative feedback and difficulties adapting to campus life may benefit greatly from a support system provided by middle-high schools and universities. This support system could include mental health education courses, psychological counselling, and other resources. By having access to such support, young students in a crucial stage of emotional and rational development can gain a systematic understanding of their own psychological state. This can help to improve students’ emotional intelligence, reduce interpersonal conflicts, and ultimately prevent the escalation of psychological pressure or mental health issues [54, 55]. To ensure the effectiveness of the support system, it is important to enhance the confidentiality and fairness of the support provided. This will encourage more young people who are experiencing psychological crises or confusion to seek help and actively engage in effective emotional counselling. By doing so, they can receive timely, customized, professional, and private support [56, 57].

Additionally, university counsellors or full-time psychological tutors should educate young students on how to efficiently manage individual stress, cope with academic setbacks and failures, and properly express their feelings and thoughts. These skills will enable them to better handle the increasing academic pressure and demands in higher grades [58].

Hypothesis five (H3) was not supported: online social support did not directly affect the mental health of university students (regardless of their gender). Related studies by Jing [59] and Zheng [60] showed that male university students with better mental health reported a preference for offline social support from family, friends, or mental health professionals rather than online social support. Specifically, participants reported that they preferred to share their confusion and thoughts about mental health-related topics and conversations in a more direct, face-to-face style. This could explain why online social support was not a critical factor in terms of directly affecting mental health among young students of different genders.

Hypothesis six (H4) was supported: online social support plays a significant moderating role in the mental health of university students, irrespective of their gender, in the face of perceived life stress. This support system effectively mitigates the impact of three specific types of perceived life stress, namely, loss, interpersonal relationships, and adaptation, on the mental well-being of male students. Similarly, online social support buffers the impact of all types of perceived life stress on female students. It can therefore be established as a protective factor and an effective strategy against various stressors for both genders. These findings align with previous research and can be attributed to the private and indirect nature of online social support. During the prolonged global epidemic, young students who experienced mental health challenges due to perceived life stress and the psychological effects of the epidemic increasingly sought support through internet platforms. The evidence supporting this claim is compelling. Fu conducted a study that showed a significant increase in the use of online mental health resources among college students during the COVID-19 pandemic [61]. A systematic review by Sharma indicated that young students turned to online platforms for support and self-help resources during the pandemic [62]. Loades reported that young people sought emotional support through various online platforms, such as social media and video calls [63]. These studies provided clear evidence of the growing reliance on internet platforms for mental health support among young students during the global epidemic. Additionally, pandemic prevention measures encouraged people to seek support online even after the spread of COVID-19 [17, 64, 65]. The private nature of online social support enables university students to seek help from online forums without inhibition. Online support has the potential to alleviate the negative impacts of challenging life events that students may face, thereby reducing the overall effect on their mental health status [11, 66]. Importantly, this support does not directly impact mental health but acts as a buffer against perceived life stress. The prolonged global epidemic significantly impacted the mental health of young students, leading to an increased need for support. Internet platforms emerged as accessible and convenient avenues for help and support during these challenging times. The prevalence of internet use among young students combined with the anonymity and accessibility of online platforms contributed to the growing reliance on internet-based resources for mental health support.

## Conclusions and implications

In summary, our study found no gender differences in overall mental health among university students. However, when examining different types of perceived life stress, we discovered that male students experienced a significantly higher impact of punishment and interpersonal relationship stress compared to females. On the other hand, female students experienced significantly higher levels of learning pressure than males.

Furthermore, specific types of perceived life stress were identified as significant predictors and risk factors for mental health among university students. For male students, the study revealed that punishment-related perceived life stress had a significant impact on their mental health, suggesting that severe perceived life stress related to punishment may worsen their mental health condition. For female students, perceived life stress related to learning pressure, interpersonal relationships, and adaptation were all identified as significant predictors and risk factors for their mental health.

Our study also uncovered both direct and moderating effects of online social support on the mental health of university students, regardless of gender, under different types of perceived life stress. This suggests that while online social support does not directly influence an individual’s mental health, it does significantly moderate it as a protective factor. Specifically, it was found to moderate the impact of loss, interpersonal relationships, and adaptation among male students, as well as all types of perceived life stress among female students.

Given the ongoing negative impact and psychological consequences of the global pandemic, the unique characteristics of online social support, such as its non-contact nature and privacy, can be utilized as a long-term and effective source of support. It can help mitigate the negative effects of various perceived life stressors and assist university students in developing a healthy and positive mindset toward the current post-COVID world, ultimately promoting their mental well-being.

This empirical study is the first to examine the mental health status and moderating effects of online social support among university students in Northwestern China under perceived life stress. It also explores gender-specific patterns within these relationships, particularly in the context of the current post-COVID world and Chinese culture. These findings contribute to existing knowledge and literature on social support systems, perceived life stress, and mental health among university students in China.

Moreover, the study highlights the importance of longitudinal efforts to promote mental health among young students worldwide. It emphasizes the significance of university mental health or counseling centers in providing necessary support through online platforms. These centers serve as crucial resources for students seeking social support regarding their stressful life experiences, mental health, or other health-related concerns, particularly in the context of the current post-pandemic world.

## 6. Limitations and future directions

The present study has certain limitations that should be acknowledged. Firstly, it should be noted that the sample used in this study was limited to students attending universities in provincial capitals and key cities. This may have resulted in the exclusion of students from county-level and township-level areas. Thus, the findings of this study may not fully generalize to the broader student population.

Secondly, this study solely relied on cross-sectional self-report measures to assess mental health, which may have limitations in capturing the complex dynamics and potential causal relationships involved, due to the limitations of our research design and available resources, we were unable to incorporate the investigation of mediating effects in our study. Meanwhile, the current study acknowledges the potential for reporting bias is crucial for maintaining scientific integrity and ensuring transparency in the study’s findings. Therefore, future research should: (1) Discuss the importance of minimizing reporting bias, explain the significance of reducing reporting bias in research and its impact on the validity and reliability of study findings, emphasize the need for future studies to address this issue diligently; (2) Consider diverse data sources and perspectives, broaden the scope of data collection by including multiple data sources and perspectives. This can help minimize the impact of potential bias arising from a single source, optimize the impact mechanism to create a more comprehensive system, and provide a more holistic understanding of the research topic. (3) Incorporate longitudinal investigations to provide a more comprehensive understanding of the factors influencing mental health patterns among Chinese university students.

Thirdly, it is important to note that mental health is a complex issue and can be affected by numerous factors, including gender. Thus it is important to approach the topic of gender differences in mental health after COVID-19 with extra caution. While it may be reasonable to state that no significant gender difference was discovered in mental health after COVID-19, it does not mean that gender has no impact on mental health during this time. Furthermore, the COVID-19 pandemic has had diverse impacts on individuals’ mental health, including increased stress, anxiety, and depression. These effects can vary based on various factors, including gender, socioeconomic status, and personal circumstances. Therefore, it is important to consider multiple factors and conduct comprehensive research to fully understand the relationship between gender and mental health during and after COVID-19 in our future study. Policy explanations should be based on solid evidence and take into account the complexity of the issue.

Fourthly, a convenience sampling strategy was employed in the present study. It is important to acknowledge that the use of convenience sampling may limit the generalizability of the findings to the broader population. Convenience sampling involves selecting participants based on their easy accessibility or proximity to the researcher, rather than employing a random or representative sampling method. As a result, the sample may not accurately represent the characteristics of the larger population, leading to potential biases and limitations in drawing conclusions. Therefore, it is necessary to interpret the findings of this study with caution and consider the potential impact of the sampling strategy on the external validity of the results.

Additionally, it is important to note that this study focused exclusively on the male/female binary when exploring mental health. However, it is increasingly recognized that transgender/gender non-conforming (TGNC) university students may face distinct challenges and higher risks of mental health problems compared to the general population [67, 68]. Despite gaining attention internationally, this topic remains largely overlooked by Chinese scholars and researchers. To address this gap, future studies should aim to include larger samples that encompass a diverse range of gender identities, which would significantly contribute to expanding knowledge in related fields.

### Electronic supplementary material

Below is the link to the electronic supplementary material.

Supplementary Material 1

## Data Availability

The data that support the findings of this study are available from School of Psychology, Shaanxi Normal University, but ethical restrictions of Shaanxi Normal University apply to the availability of these data, which contains privacy variables that might affect the growth of adolescents’ mental health and were used under license for the current study, and so are not publicly available. Data are however available from the corresponding author upon reasonable request and with permission of School of Psychology, Shaanxi Normal University.
