# Supplementary material for: The impact of perceived life stress and online social support on university students’ mental health during the post-COVID era in Northwestern China: gender-specific analysis
Source: BMC Public Health. 2024 Feb 14;24:467. doi: 10.1186/s12889-024-17935-x (PMC10868037; doi:10.1186/s12889-024-17935-x)
Supplement: Supplementary file 1 — Supplementary Material 1 [file 12889_2024_17935_MOESM1_ESM.pdf]

Editing Certificate

This document certifies that the manuscript

The impact of perceived life stress and online social support on university students' mental health during the Post-COVID Era in Northwestern China: Gender - specific analysis

prepared by the authors

Moye Xin, Chengxi Yang, Lijin Zhang, Chenzhuo Gao, Sasa Wang

was edited for proper English language, grammar, punctuation, spelling, and overall style by one or more of the highly qualified native English speaking editors at AJE.

This certificate was issued on **December 18, 2023** and may be verified on the AJE website using the verification code **9C93-FD03-628B-AFC1-E7B8**.

Neither the research content nor the authors' intentions were altered in any way during the editing process. Documents receiving this certification should be English-ready for publication; however, the author has the ability to accept or reject our suggestions and changes. To verify the final AJE edited version, please visit our verification page at aje.com/certificate. If you have any questions or concerns about this edited document, please contact AJE at support@aje.com.

AJE provides a range of editing, translation, and manuscript services for researchers and publishers around the world.
For more information about our company, services, and partner discounts, please visit aje.com.
